# Association of interleukin-10 gene polymorphisms with breast cancer in a Chinese population

**DOI:** 10.1186/1756-9966-29-72

**Published:** 2010-06-17

**Authors:** Fanjun Kong, Jie Liu, Yongheng Liu, Bao Song, Hualing Wang, Wenchao Liu

**Affiliations:** 1 Department of Oncology, State Key Discipline of Cell Biology, Xijing Hospital, Fourth Military Medical University, 710032, Xi'an, PR China; 2 Department of Oncology, the 456 Hospital of Chinese PLA, 250031, Jinan, PR China; 3 Department of Oncology, Shandong Cancer Hospital & Institute, 250031, Jinan, PR China; 4 Department of Oncology, Guang'anmen Hospital, China Academy of Chinese Medical Sciences, 100053, Beijing, PR China; 5 Department of Basic Research Center, Shandong Cancer Hospital & Institute, 250031, Jinan, PR China

## Abstract

**Backgroud:**

Interleukin-10(IL-10) is a multifunctional cytokine with both immunosuppressive and antiangiogenic functions. Polymorphisms in the IL-10 gene promoter genetically determine interindividual differences in IL-10 production. This study was performed to determined whether polymorphisms in the IL-10 gene promoter were associated with breast cancer in a Chinese Han population.

**Methods:**

We genotyped 315 patients with breast cancer and 322 healthy control subjects for -1082A/G, -819T/C and -592A/C single nucleotide polymorphisms in the promoter region of the IL-10 gene by polymerase chain reactionerestriction fragment length polymorphism (PCR-RFLP).

**Results:**

There were no significant differences in genotype, allele, or haplotype frequencies in all three loci between patients and healthy controls. Analysis of breast cancer prognostic and predictive factors revealed that the -1082AA genotype was associated with a significantly increased risk of lymph node (LN) involvement (*P *= 0.041) and larger tumor size (*P *= 0.039) at the time of diagnosis. Furthermore, in the haplotype analysis of IL-10 gene, we found that patients carrying ATA haplotype were in higher LN involvement (*p *= 0.022) and higher tumor stage(*p *= 0.028) of breast cancer at the time of diagnosis compared with others.

**Conclusions:**

Our findings suggest that IL-10 promoter polymorphisms participate in the progression of breast cancer rather than in its initial development in Chinese Han women.

## Introduction

Breast cancer is the most common malignancy threatening the health and life of women and it's incidence has increased in recent years in both developed and developing countries[1]. Biologic mechanisms leading to the development of breast cancer are not clearly understood, but the role of cytokines in cancer immunity and carcinogenesis has been well established[2]. As a multifunctional Th2-cytokine with both immunosuppressive and anti-angiogenic functions, interleukin-10 (IL-10) may have both tumor-promoting and tumor-inhibiting properties[3]. Recent data suggest that polymorphic variations in the promoter sequences of IL-10 gene may influence the gene expression[4,5] and consequently play a certain role in susceptibility and clinical course of breast cancer.

IL-10 is an important immunoregulatory cytokine mainly produced by activated T cells, monocytes, B cells and thymocytes. As an immune response modulator, IL-10 can both stimulate and suppress the immune response[6]. Numerous studies have shown that IL-10 may be involved in the pathogenesis of cancer, but the results were inconsistent. On the one hand, increased serum IL-10 levels could facilitate development of cancer by suppressing expression of MHC class I and II antigens[7] and preventting tumor antigen presentation to CD8-cytotoxic T lymphocytes. On the other hand, anti-angiogenic effects of IL-10 are supposed to play a protective and preventive role against tumor.

The gene encoding IL-10 is located on human chromosome 1q31-1q32[8,9], and is composed of five exons and four introns. It has been reported that several important polymorphic sites in the IL-10 gene, including three in the promoter region (-1082 (A/G, -819 T/C, -592 A/C) may influence the transcription of IL-10 messenger RNA and the expression of IL-10 in vitro [10-12]. Although several studies have shown the possible involvement of IL-10 in the pathogenesis of breast cancer, as well as its association with prognosis in different ethnic populations, the results were not all consistent[13]. Furthermore, little is known about the effect of these polymorphisms on the risk of beast cancer in the Han Chinese population. The goal of this study was to evaluate whether IL-10 gene promoter -1082A/G, -819T/C and -592A/C polymorphisms and haplotypes were associated with breast cancer in a Han Chinese population.

## Materials and methods

### Subjects

Blood samples were taken from 315 breast cancer cases and 322 non-cancer controls. The case group was recruited between October 2008 and October 2009 from the Shan Dong Cancer Hospital and the PLA 456 Hospital. The patients' pathological and clinical information were obtained from their medical files. All cases were newly diagnosed and previously untreated. The control group consisted of 322 healthy age-matched women who visited the general health check-up division at the two hospitals in the period between October 2008 and October 2009. Selection criteria for controls were no evidence of any personal or family history of cancer or other serious illness. At recruitment, each participant was personally interviewed to obtain detailed information on demographic characteristics and lifetime history of tobacco and alcohol use. All subjects were unrelated ethnic Han Chinese and residents of northern China. The study has been approved by the Institutional Review Boards of Shan Dong Cancer Hospital and the PLA 456 Hospital. Written informed consent was obtained from all participating subjects.

### Polymorphism analysis

Genomic DNA was isolated from peripheral blood leukocytes of control subjects and breast cancer patients by the salting-out method as described previously [14]. Genotypes were assayed with polymerase chain reactionerestriction fragment length polymorphism(RFLP) methods. The PCR primers were designed based on described previously[15]. The PCR was performed with a 25-μL reaction mixture containing 100 ng of genomic DNA, 0.5 μmol/L of each primer, 200 μmol/L of each dNTP, 2.5U of Taq DNA polymerase (Omega, Doraville, GA), 10× PCR buffer supplied by Invitrogen Corp (10 mmol/l Tris-HCl, pH 8.8, 50 mmol/l KCl), and 2.0 mmol/L MgCl_2_. The PCR profile consisted of an initial melting step of 5 minutes at 94°C, followed by 35 cycles of 30 seconds at 94°C, 45 seconds at 58°C for -1082A/G, 59°C for -819 T/C and 62°C for -592 A/C; 55 s at 72°C; and a final elongation at 72°C for 8 min. The restriction endonucleases MnlI, MaeIII, and RsaI (New England Biolabs, Beverly, MA) were used to distinguish the IL-10 gene -1082A/G, -819T/C, -592A/C polymorphisms, respectively (Table1). To confirm the genotyping results, PCR-amplified DNA samples were examined by DNA sequencing, and the results were 100% concordant (data not shown).

**Table 1:** Primer sequences and reaction conditions for genotyping IL-10 polymorphisms

Polymorphism	db SNP ID	PCR Primer sequence	RE	Product size(bp)
-1082 A/G	Rs1800870	F: 5'-CTCGCTGCAACCCAACTGGC-3' R: 5'-TCTTACCTATCCCTACTTCC-3'	MnlI	G: 106+33 A: 139
-819 T/C	Rs1800871	F: 5-TCATTCTATGTGCTGGAGATGG-3' R: 5'-TGGGGGAAGTGGGTAAGAGT-3'	MaeIII	C:125+84 T: 209
-592 A/C	Rs1800872	F: 5-GGTGAGCACTACCTGACTAGC-3' R: 5'-CCTAGGTCACAGTGACGTGG-3'	RsaI	A:236+176 C: 412

Abbreviations: dbSNP ID, database identifier; SNP, single-nucleotide polymorphism; PCR, polymerase chain reaction; RE, restriction endonuclease.

### Statistical analysis

Genotype and allele frequencies of IL-10 were compared between breast cancer cases and controls by the chi-squared test or Fisher's exact test when necessary. The odds rations (OR) and 95% confidence interval (CI) were calculated to estimate the relative risk conferred by a particular allele and genotype. Demographic and clinical data between groups were compared by chi-squared test and by Student's t-test. Statistical significance was assumed at the *p *< 0.05 level. The SPSS for Windows (version 13.0; SPSS, Inc) was used for all of the statistical analysis.

## Results

### Subject characteristics

The demographics of the cases and controls enrolled in this study are summarized in Table2. There were no statistically significant differences between the cases and controls for the age, menopausal status (*P *= 0.979 and *P *= 0.593, respectively), and this suggested that the matching based on these two variables was adequate.

**Table 2:** Characteristics of patients with breast cancer and healthy controls

Variable	Patients, no. (%)	Controls, no. (%)	*P-*value
		
	n = 315	n = 322	
Age(year)			0.979
< 48	165 (52.4)	169 (52.5)	
≥48	150 (47.6)	153 (47.5)	
Menopausal status			0.593
Premenopausal	144 (45.7)	154 (47.8)	
Postmenopausal	171 (54.3)	168 (52.2)	
Tumor size (cm)			
< 2	104 (33.0)		
2~5	167 (53.0)		
≥5	44 (14.0)		
LN involvement			
Positive	117 (37.1)		
Negative	198 (62.9)		
ER expression			
Positive	169 (53.7)		
Negative	146 (46.3)		
PR expression			
Positive	166 (52.7)		
Negative	149 (47.3)		

### Genotype and allele frequencies

The genotype and allele frequencies of the IL-10 gene polymorphisms in breast cancer patients and healthy controls are show in Table3. The genotypes were found to be in Hardy-Weinberg equilibrium in both case and control groups. Statistical analysis, however, revealed no significant differences in the genotype and allele frequencies at all three SNP sites between patients and healthy controls. In addition to overall comparisons, the genotype frequencies were compared in subgroups classified according to menopausal status and no association was found between genotypes and risk of breast cancer.

**Table 3:** Genotype and allele frequencies of IL-10 promoter polymorphisms in breast cancer patients and healthy controls

	Frequency, no.(%)				Frequency, no.(%)		
					
Genetype	Patients n = 315	Controls n = 322	*P*-value	Alleles	Patients 2n = 630	Controls 2n = 644	*P*-value
-1082 A/G			0.664	-1082 A/G			0.374
AA	285 (90.5)	285 (88.5)		A	599 (95.1)	605 (93.9)	
AG	29 (9.2)	35 (10.9)		G	31 (4.9)	39 (6.1)	
GG	1 (0.3)	2 (0.6)					
-819 T/C			0.604	-819 T/C			0.315
TT	119 (37.8)	134 (41.6)		T	373 (59.2)	399 (62.0)	
TC	135 (42.9)	131 (40.7)		C	257 (40.8)	245 (38.0)	
CC	61 (19.3)	57 (17.7)					
-592 A/C			0.604	-592 A/C			0.315
AA	119 (37.8)	134 (41.6)		A	373 (59.2)	399 (62.0)	
AC	135 (42.9)	131 (40.7)		C	257 (40.8)	245 (38.0)	
CC	61 (19.3)	57 (17.7)					

Analysis of association between genotypes and clinicopathologic features of breast cancer revealed no association between genotypes at these positions and ER expression and PR expression. Tumor size was significantly different between carriers of the AA genotype at position -1082 A/G in comparison to other genotypes (*P *= 0.039). In addition, LN involvement was significantly lower in patients harboring at least one G allele at position -1082 A/G (AG and GG genotypes) in comparison to patients with the AA genotype(*P *= 0.041). (Table4)

**Table 4:** Genotype frequencies of IL-10 and clinicopathologic features of breast cancer patients

Clinicopathologic features	n	Genetype (%)		*χ*^2^	*p*
				
		AA	AG+GG		
ER expression				0.001	0.971
Positive	169	153 (90.5)	16 (9.5)		
Negative	146	132 (90.4)	14 (9.6)		
PR expression				0.209	0.647
Positive	166	149 (89.8)	17 (10.2)		
Negative	149	136 (91.3)	13 (8.7)		
Tumor size (cm)				6.471	0.039
< 2	104	88 (84.6)	16 (15.4)		
2~5	167	155 (92.8)	12 (7.2)		
≥5	44	42 (95.5)	2 (4.5)		
LN involvement				4.174	0.041
Negative	198	174 (87.9)	24 (12.1)		
Positive	117	111 (94.9)	6 (5.1)		

### Haplotypes analysis

The estimated haplotype frequencies of IL-10 polymorphisms in breaste cancer patients and controls are shown in Table5. Complete linkage disequilibrium was observed between locus -819T/C and locus -592 A/C. Four possible haplotypes were demonstrated in our population. The most frequent haplotype in both patients and controls was ATA haplotype(harboring wild type alleles of all three positions and with 56.5% frequency in patients vs. 58.5% in controls). The frequencies of haplotype were investigated and no significant differences were observed between patients and healthy controls.

**Table 5:** Frequencies of IL-10 Haplotypes(-1082, -810, -592) in breast cancer patients and healthy controls

	Patients, no. (%)	Controls, no. (%)		
			
Possible haplotype	2n = 630	2n = 644	*χ*^2^	*P*-value
ATA	356 (56.5)	377 (58.5)	1.857	0.603
ACC	243 (38.6)	228 (35.4)		
GTA	17 (2.7)	22 (3.4)		
GCC	14 (2.2)	17 (2.6)		

Analysis of breast cancer prognostic and predictive factors revealed that ATA haplotype was associated with a significantly increased risk of lymph node metastasis at the time of diagnosis as compared with other haplotypes(*P *= 0.022). In addition, we also found strong association between tumor size and the ATA haplotypes(*P *= 0.028). (Table6)

**Table 6:** Frequencies of IL-10 Haplotypes(-1082, -810, -592) and clinicopathologic features of breast cancer patients

		haplotype (%)			
				
Clinicopathologic features	2n	ATA	non-ATA	*χ*^2^	*p*
ER expression				0.026	0.872
Positive	338	192 (56.8)	146 (43.2)		
Negative	292	164 (56.2)	128 (43.8)		
PR expression				0.010	0.922
Positive	332	187 (56.3)	145 (43.7)		
Negative	298	169 (56.7)	129 (43.3)		
Tumor size (cm)				7.180	0.028
< 2	208	105 (50.5)	103 (49.5)		
2~5	334	192 (57.5)	142 (42.5)		
≥5	88	59 (67.0)	29 (33.0)		
LN involvement				5.246	0.022
Negative	396	210 (53.0)	186 (47.0)		
Positive	234	146 (62.4)	88 (37.6)		

## Discussion

In this case-control study we evaluated the association between the polymorphisms of the IL-10 promoter and breast cancer in a Han Chinese population. Our data did not show significant differences in allele, genotype and haplotype frequencies between breast cancer patients and healthy controls. In concordance with our study, Howell et al.[16], Smith et al. [17] and Balasubramanian et al.[18] reported that there were no apparent relationship of the IL-10 gene promoter polymorphisms with the risk of breast cancer. However, these results are not consistent with the study conducted in Austrian previously, in which the -592AA genotype was shown to be associated with a reduced breast cancer risk[19]. Moreover, another study from the Italian population showed that the IL-10 -1082AA genotype was correlated with a marked increase in breast cancer risk[20]. Although it is difficult to determine the reasons behind the contradictory results in these studies, the different genetic background of study population may be one of the main factors.

In this study, we found that the frequencies of the -1082 G, -819 C and -592 C alleles among the healthy controls (0.061, 0.380 and 0.380, respectively) were similar to those observed in an Asian population[21,22] but significantly lower than those of European Caucasians [23-25]. We also found that there was strong linkage disequilibrium among the -1082A/G, -819 T/C and -592 A/C polymorphisms. Complete linkage disequilibrium was observed between locus -819T/C and locus -592 A/C. Four possible haplotypes were demonstrated in our population. Major haplotype frequency of the ATA among the controls in the present study was 0.585, which was significantly higher than those of study performed in the European Caucasians (0.290 and 0.248, respectively)[24,26]. These results suggest that the frequencies of IL-10 gene alleles and haplotypes might vary among the different ethnic population.

Although we did not find an association of the IL-10 gene polymorphisms with risk of breast cancer, in present study we reported for the first time that the IL-10 promoter haplotypes and -1082 A/G polymorphism were significantly associated with the prognostic and predictive factors of breast cancer in Chinese han women. Our data showed that the -1082AA genotype was associated with a significantly increased risk of lymph node (LN) involvement (*P *= 0.041) and larger tumor size (*P *= 0.039) at the time of diagnosis. Moreover, in the haplotype analysis of IL-10 gene, we also found that patients carrying ATA haplotype were in higher LN involvement (*p *= 0.022) and higher tumor stage(*p *= 0.028) of breast cancer at the time of diagnosis compared with others. The findings suggest that the IL-10 ATA haplotype and -1082AA genotype might be adverse prognostic factors in breast cancer in Chinese Han women.

IL-10 is a multifunctional cytokine with both immunosuppressive and antiangiogenic functions, which may play varied roles in the pathogenesis and development of breast cancer. Although the genetic control of IL-10 expression is not clearly understood yet, polymorphisms in promoter have been reported to determine interindividual differences in IL-10 production[4,9]. Previous studies indicated that IL-10 promoter -1082 AA genotype was associated with decreased IL-10 expression[27]. ATA haplotype formed by polymorphisms at positions -1,082, -819 and -592 in the promoter of the IL-10 gene has been is generally assumed to be a lower IL-10 responder[10-12]. This and the present study indicate that low levels of IL-10 may play a facilitative role in the development of breast cancer. Findings of our study are further supported by a recent study showing that the low-expression allele and haplotype were associated with reduced disease-free survival and the IL-10 gene polymorphisms may be a potential prognosis marker in breast cancer for disease-free survival[28]. The mechanism for this remains unclear, but may likely include anti-angiogenic functions of IL-10.

In conclusion, in this case-control study, we report for the first time that the IL-10 promoter polymorphisms were significantly associated with the prognostic and predictive factors of breast cancer in a Chinese han population. The main finding of our study suggests that IL-10 promoter polymorphisms participate in the progression of breast cancer rather than in its initial development. Considering limited sample size, nonrandom sampling and pitfalls of unknown confounders, further studies with larger sample size from different ethnic origins are required to confirm and extend our observations. In addition, more studies should be carried out to clarify the exact molecular mechanism of IL-10 polymorphisms effects.

## Competing interests

The authors declare that they have no competing interests.

## Authors' contributions

WL, FK and JL designed the study, collected the materials, performed all experiments, YL drafted the manuscript. BS and HW participated in the study and performed the statistical analysis. All authors read and approved the final version manuscript.

## References

[B1] Kamangar F Dores GM Anderson WF Patterns of cancer incidence, mortality, and prevalence across five continents:defining priorities to reduce cancer disparities in different geographic regions of the world J Clin Oncol 2006 24 14 2137 2150 10.1200/JCO.2005.05.2308 16682732

[B2] Smyth MJ Cretney E Kershaw MH Hayakawa Y Cytokines in cancer immunity and immunotherapy Immunol Rev 2004 202 275 293 10.1111/j.0105-2896.2004.00199.x 15546400

[B3] Mocellin S Marincola F Rossi CR Nitti D Lise M The multifaceted relationship between IL-10 and adaptive immunity:putting together the pieces of a puzzle Cytokine Growth Factor.Rev 2004 15 61 76 10.1016/j.cytogfr.2003.11.001 14746814

[B4] Turner DM Williams DM Sankaran D Lazzarus M Sinnott PJ Hutchinson IV An investigation of polymorphism in the interleukin-10 gene promoter Eur J Immunogenet 1997 24 1 8 9043871 10.1111/j.1365-2370.1997.tb00001.x

[B5] Gibson AW Edberg JC Wu J Westendorp RG Huizinga TW Kimberly RP Novel single nucleotide polymorphisms in the distal IL-10 promoter affect IL-10 production and enhance the risk of systemic lupus erythematosus J Immunol 2001 166 3915 3922 11238636 10.4049/jimmunol.166.6.3915

[B6] Mocellin S Marincola FM Young HA Interleukin-10 and the immune response against cancer: a counterpoint J Leukoc Biol 2005 78 1043 1051 10.1189/jlb.0705358 16204623

[B7] Matsuda M Salazar F Petersson M Masucci G Hansson J Pisa P Interleukin 10 pretreatment protects target cells from tumor- and allospecific cytotoxic T cells and downregulates HLA class I expression J Exp Med 1994 180 2371 2376 10.1084/jem.180.6.2371 7964510 PMC2191780

[B8] Kim JM Brannan CI Copeland NG Structure of the mouse IL-10 gene and chromosomal localization of the mouse and human genes J Immunol 1992 148 3618 3623 1350294

[B9] Eskdale J Kube D Tesch H Mapping of the human IL10 gene and further characterization of the 5'flanking sequence Immunogenetics 1997 46 120 128 10.1007/s002510050250 9162098

[B10] Kingo K Ratsep R Koks S Karelson M Silm H Vasar E Influence of genetic polymorphisms on interleukin-10 mRNA expression and psoriasis susceptibility J Dermatol Sci 2005 37 111 113 10.1016/j.jdermsci.2004.10.002 15659329

[B11] Crawley E Kay R Sillibourne J Patel P Hutchinson I Woo P Polymorphic haplotypes of the interleukin-10 5'flanking region determine variable interleukin-10 transcription and are associated with particular phenotypes of juvenile rheumatoid arthritis Arthritis Rheum 1999 42 1101 1108 10.1002/1529-0131(199906)42:6<1101::AID-ANR6>3.0.CO;2-Y 10366102

[B12] Hoffmann SC Stanley EM Darrin E Cox Craighead N DiMercurio BS Koziol DE Harlan DM Kirk AD Blair PJ Association of cytokine polymorphic inheritance and in vitro cytokine production in anti-CD3/CD28-stimulated peripheral blood lymphocytes Transplantation 2001 72 1444 1450 10.1097/00007890-200110270-00019 11685118

[B13] Howell WM Rose-Zerilli MJ Cytokine gene polymorphisms, cancer susceptibility, and prognosis J Nutr 2007 137 1 Suppl 194S 199S 17182825 10.1093/jn/137.1.194S

[B14] John SW Weitzner G Rozen R Scriver CR A rapid procedure for extracting genomic DNA from leukocytes Nucleic Acids Res 1991 19 408 10.1093/nar/19.2.408 2014181 PMC333617

[B15] Shih CM Lee YL Chiou HL The involvement of genetic polymorphism of IL-10 promoter in non-small cell lung cancer Lung Cancer 2005 50 3 291 297 10.1016/j.lungcan.2005.07.007 16122836

[B16] Howell WM Rose-Zerilli MJ Interleukin- 10 polymorphisms, cancer susceptibility and prognosis Fam Cancer 2006 5 2 143 149 10.1007/s10689-005-0072-3 16736283

[B17] Smith KC Bateman AC Fussell HM Howell WM Cytokine gene polymorphisms and breast cancer susceptibility and prognosis Eur J Immunogenet 2004 31 167 173 10.1111/j.1365-2370.2004.00462.x 15265021

[B18] Balasubramanian SP Azmy IA Higham SE Interleukin gene polymorphisms and breast cancer: a case control study and systematic literature review BMC Cancer 2006 6 188 10.1186/1471-2407-6-188 16842617 PMC1553474

[B19] Langsenlehner U Krippl P Renner W Yazdani-Biuki B Eder T Koppel H Wascher TC Paulweber B Samonigg H Interleukin- 10 promoter polymorphism is associated with decreased breast cancer risk Breast Cancer Res Treat 2005 90 113 5 10.1007/s10549-004-3607-7 15803357

[B20] Giordani L Bruzzi P Lasalandra C Quaranta M Schittulli F Della F Ragione Iolascon A Polymorphisms of Interleukin-10 and tumour necrosis factor-α gene promoter and breast cancer risk Clin Chem 2003 49 1664 1667 10.1373/49.10.1664 14500594

[B21] Meenagh A Willians F Ross OA Frequency of cytokinepolymor phisms in populations from western Europe, Africa, Asia, the Middle East and South America Human Immunol 2002 63 1055 1061 10.1016/S0198-8859(02)00440-8 12392859

[B22] Chin HJ Na KY Kim SJ Interleukin- 10 promoter polymorphism is associated with the predisposition to the development of IgA nephropathy and focal segmental glomeruloselerosis in Korea J Korean Med Sci 2005 20 6 989 993 10.3346/jkms.2005.20.6.989 16361810 PMC2779332

[B23] Alonso R Suarez A Castro P Lacave AJ Gutierrez C Influence of interleukin-10 genetic polymorphism on survival rates in melanoma patients with advanced disease Melanoma Res 2005 15 53 60 10.1097/00008390-200502000-00009 15714121

[B24] Scassellati C Zanardini R Squitti R Promoter haplotypes of interleukin-10 gene and sporadic Alzheimer's disease Neurosci Lett 2004 35 119 122 10.1016/j.neulet.2003.11.033 14746878

[B25] Poli F Nocco A Berra S Allelle frequencies of polymorphisms of TNFα, IL-6, IL-10 and IFN G in an Italian Caucasian population Eur J Immunogrnet 2002 29 3 237 240 10.1046/j.1365-2370.2002.00303.x 12047360

[B26] Mangia A Santoro R Piattelli M IL- 10 haplotypes as possible predictors of spontaneous clearance of HCV infection Cytokine 2004 25 103 109 10.1016/j.cyto.2003.10.005 14698136

[B27] Eskdale J Gallagher A polymorphic dinucleotide repeat in the human IL-10 promoter Immunogenetics 1995 42 444 445 10.1007/BF00179416 7590988

[B28] Gerger A Renner W Langsenlehner T Hofmann G Knechtel G Szkandera J Samonigg H Krippl P Langsenlehner U Association of interleukin-10 gene variation with breast cancer prognosis Breast Cancer Res Treat 2010 119 701 705 10.1007/s10549-009-0417-y 19437115

